# Supplementary material for: Contrasting Asymptomatic and Drug Resistance Gene Prevalence of Plasmodium falciparum in Ghana: Implications on Seasonal Malaria Chemoprevention
Source: Genes (Basel). 2019 Jul 16;10(7):538. doi: 10.3390/genes10070538 (PMC6678124; doi:10.3390/genes10070538)
Supplement: Supplementary file 1 [file genes-10-00538-s001.pdf]

Supplementary Materials: Contrasting Asymptomatic and Drug Resistance Gene Prevalence of *Plasmodium falciparum* in Ghana: Implications on Seasonal Malaria Chemoprevention

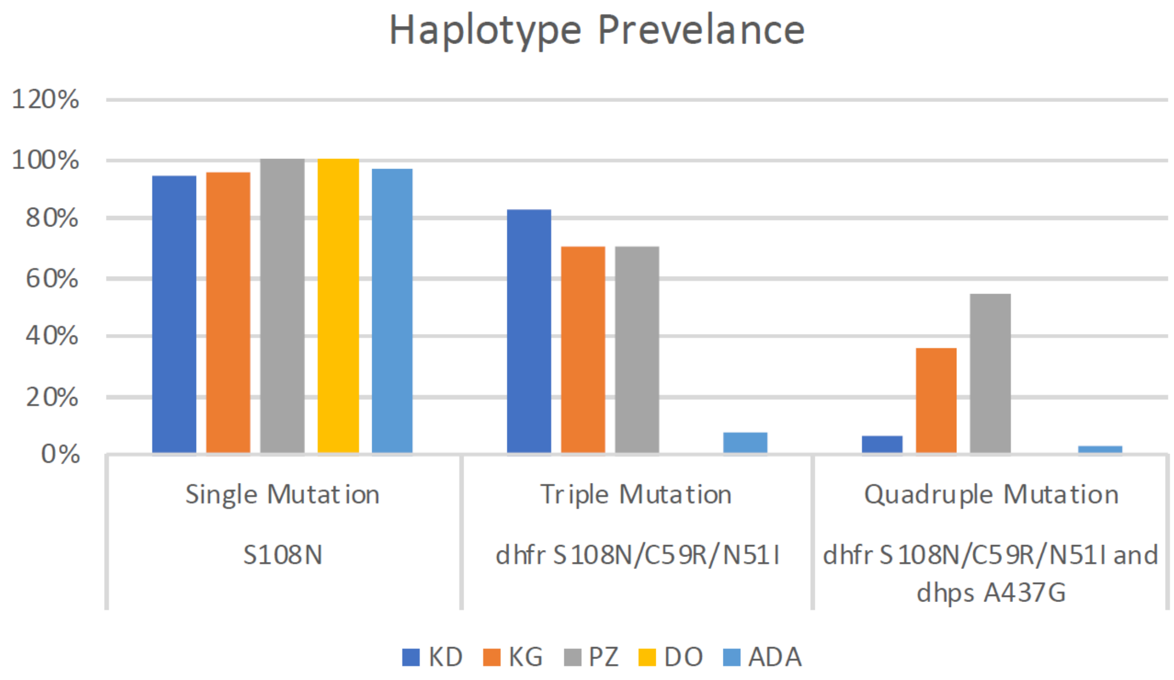

Figure S1. Percentage of single, triple, and quadruple mutations of *pfdhfr* and *pfdhps* among *P. falciparum* samples from respective sites in Ghana.

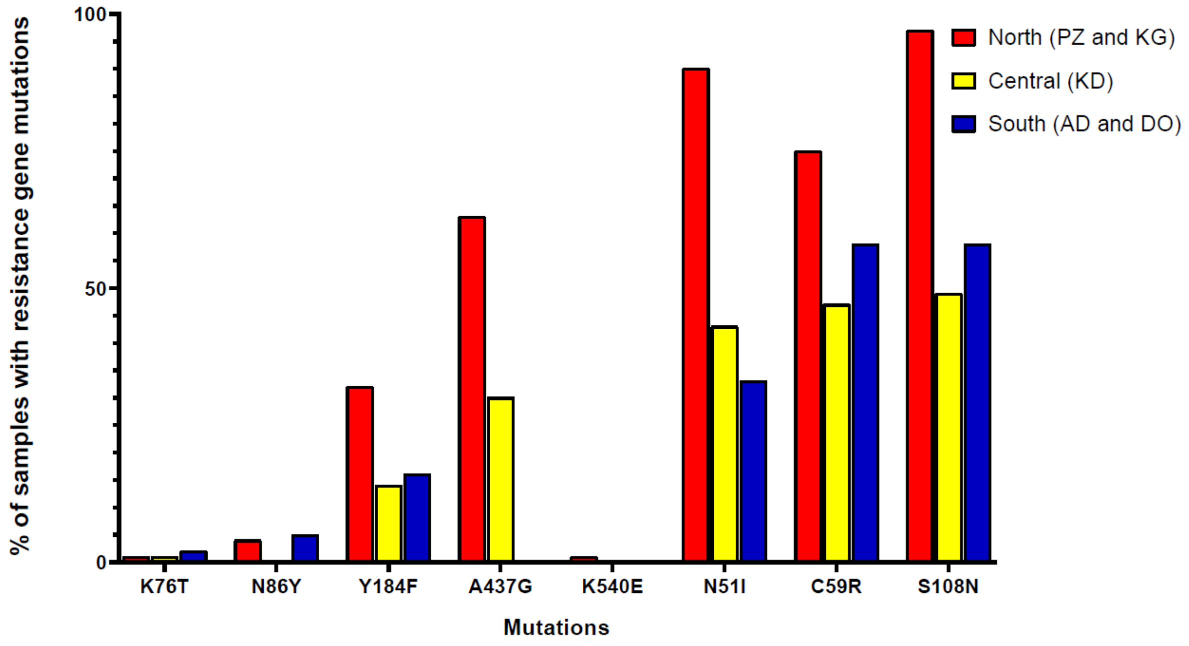

Figure S2. Percentage of *P. falciparum* samples that showed mutations in the respective resistance gene codons.

Table S1. Primers and PCR conditions for *P. falciparum* resistance gene amplifications.

Primers	Sequence<br>F 5'>3'<br>R 5'>3'	Expected Band Size	PCR Conditions	Reference
Genes related to AQ (amodiaquine) and CQ (chloroquine) resistance				
crt-K76T	GGCTCACGTTTAGGTGGA<br>TGAATTTCCCTTTTTATTTCCAAA	264 bp	95°C for 5:00<br>95°C for 00:30<br>50°C for 01:30<br>72°C for 01:30<br>Go to step2 34 times<br>72°C for 5:00<br>10°C for infinite	Vathsala et al 2004
mdr1-N86Y	ATGGGTAAAGAGCAGAAAGA<br>AACGCAAGTAATACATAAAGTCA	603 bp	95°C for 5:00<br>95°C for 00:30<br>52°C for 01:30<br>72°C for 01:30<br>Go to step2 34 times<br>72°C for 5:00<br>10°C for infinite	Vathsala et al 2004
Genes related to SP (sulfadoxine-pyrimethamine) resistance				
dhfr-nested2a	TTTATGATGGAACAAGTCTGCGACGTT<br>AAATTCTTGATAAACAACGGAACCTttTA	450 bp	94°C for 3:00<br>94°C for 1:00<br>57°C for 01:00<br>72°C for 01:00<br>Go to step2 39 times<br>72°C for 10:00<br>10°C for infinite	Duraisingh et al 1998
dhps-nested1	AACCTAAACGTGCTGTTCAA<br>AATTGTGTGATTTGTCCACAA	600 bp	95°C for 5:00<br>95°C for 00:30<br>52°C for 01:30<br>72°C for 01:30<br>Go to step2 34 times<br>72°C for 5:00<br>10°C for infinite	Duraisingh et al 1998
Sensitivity and Specificity				
PCR 18s	TTAAACTGGTTTGGGAAAACCAAATATATT<br>ACACAATGAACTCAATCATGACTACCCGTC	206	95°C for 5:00<br>95°C for 00:30<br>58°C for 01:30<br>72°C for 01:00<br>Go to Step2 35 times<br>72°C for 05:00	Wang et al 2014
qPCR 18s	AGTCATCTTTCGAGGTGACTTTTAGATTGCT<br>GCCGCAAGCTCCACGCCTGGTGGTGC	-	95°C for 03:00<br>94°C for 00:30<br>68°C for 01:00<br>95°C for 00:10<br>Got to step2 45 times<br>**melting curve from 65°C to 95°C with 0.5°C increments for 00:05	Baum et al 2015
SYBR-TARE	ctatgttgcacttacatgcayaat<br>tgacctaagaagtavaataatgatga	-	50°C for 2:00<br>95°C for 10:00<br>95°C for 00:15<br>57°C for 01:00<br>Go to Step2 45 times<br>10°C for infinite	Hofmann et al 2014

Table S2. Information of *P. falciparum* samples collected in each of the study sites in Ghana.

SITE	SEASON	NO. OF CHILDREN SCREENED	Microscopy Positives (Prevalence %)	NESTED 18s PCR Positives (Prevalence %)
KONONGO	WET	216	52 (24.07%)	60 (27.78%)
ADA	WET	148	19 (12.84%)	25 (16.89%)
KUMBUNGU,KPASOLGU, NAVRONGO	WET	65	12 (18.46%)	25 (38.46%)
PAGAZA,TAMALE	WET	47	12 (25.53%)	33 (70.21%)
DODOWA	WET	59	10 (16.95%)	10 (16.95%)
**Total Number of Positives**		535	105	153
